# Efficient Streaming Mass Spatio-Temporal Vehicle Data Access in Urban Sensor Networks Based on Apache Storm

**DOI:** 10.3390/s17040815

**Published:** 2017-04-10

**Authors:** Lianjie Zhou, Nengcheng Chen, Zeqiang Chen

**Affiliations:** 1 State Key Laboratory of Information Engineering in Surveying, Mapping, and Remote Sensing, Wuhan University, Luoyu Road 129, Wuhan 430079, China; zlj0808@whu.edu.cn; 2 Collaborative Innovation Center of Geospatial Technology, 129 Luoyu Road, Wuhan 430079, China; ZeqiangChen@whu.edu.cn

**Keywords:** BeiDou bus network, streaming spatio-temporal mass data access, Apache Storm, Sensor Observation Service, cloud computing

## Abstract

The efficient data access of streaming vehicle data is the foundation of analyzing, using and mining vehicle data in smart cities, which is an approach to understand traffic environments. However, the number of vehicles in urban cities has grown rapidly, reaching hundreds of thousands in number. Accessing the mass streaming data of vehicles is hard and takes a long time due to limited computation capability and backward modes. We propose an efficient streaming spatio-temporal data access based on Apache Storm (ESDAS) to achieve real-time streaming data access and data cleaning. As a popular streaming data processing tool, Apache Storm can be applied to streaming mass data access and real time data cleaning. By designing the Spout/bolt workflow of topology in ESDAS and by developing the speeding bolt and other bolts, Apache Storm can achieve the prospective aim. In our experiments, Taiyuan BeiDou bus location data is selected as the mass spatio-temporal data source. In the experiments, the data access results with different bolts are shown in map form, and the filtered buses’ aggregation forms are different. In terms of performance evaluation, the consumption time in ESDAS for ten thousand records per second for a speeding bolt is approximately 300 milliseconds, and that for MongoDB is approximately 1300 milliseconds. The efficiency of ESDAS is approximately three times higher than that of MongoDB.

## 1. Introduction

Currently, vehicle observation streaming mass data access is a research hotspot in smart cities [1,2,3]. When the size of vehicles is n, the frequency of streaming data access is n per second. With the rapid increase in the number of vehicles in urban cities, the frequency of data access is raised exponentially. Sensor networks produce continuous real-time and time-ordered data, called streaming data [4]. As analyzed by Choong, the characteristics of streaming data are unbounded data size, structured or semi-structured, time ordered and an unfixed data unit [5]. Along with the application of sensor data, the study of processing sensor data requires more work. The study of streaming data access has great practical value. Streaming data appears regularly in our lives, such as in cell phone positioning, video surveillance, and air sensor monitoring so on. Accessing the streaming data is difficult, so the efficient real-time processing of data is hard to achieve [6]. As the number of sensors increases, the efficient real-time processing of data becomes more difficult [7].

Currently, there are several vehicle monitoring networks, for example, the Shenzhen taxi network, the Wuhan taxi network, the Taiyuan bus network and the New York taxi network. The number of vehicles in the Shenzhen taxi network is 25,000, the number of vehicles in Wuhan is 12,000, the number of vehicles in Taiyuan is 2300 and the number of vehicles in New York is 33,000. Vehicles locate and send a message every 60 s in the Shenzhen taxi network and every 30 s in the Wuhan taxi network, Taiyuan bus network and New York taxi network. Moreover, the numbers of vehicles in these sensor networks are growing at a certain rate. To access the streaming data of taxis or buses, there are many techniques available, such as high-performance databases, cache mechanisms, distributed data storage centers and other efficient access algorithms [8,9,10].

The mass data access methods can be divided into direct data storage methods and indirect data filtering methods. In direct data storage methods, a distributed database is provided with good performance, substantial storage, high scalability and high availability characteristics. MongoDB [11], Redis [12], HBase [13] and Cassandra [14] are widely used distributed databases, where MongoDB is a lightweight, stable and easy to recover and high performance database [15]. In indirect data filtering methods, Lin proposed two strongly consistent data access algorithms, including poll-each-read and callback [16]. Kumar proposed a new proxy level web caching mechanism that takes into account the aggregate patterns observed in user object requests [17]. Tian proposed a novel probabilistic caching mechanism for media on-demand systems based on peer-to-peer with less workload imposed on the server [18]. However, these streaming data access methods cannot fully support the massive number of sensors, such as the one hundred thousand level number of sensors. The frequency of data access per second is approximately 60 thousand in the Taiyuan BeiDou (BD) bus network. MongoDB supports the data insertion capability of 300 milliseconds with sixty thousand records per second with five shards in superior configuration. When meeting the data frequency of one hundred and twenty thousand records per second, MongoDB is unable to function properly. The whole time of such a size of data access is approximately one hour or endless, as it is unable to handle such a volume of sensor data. In addition, there is valuable information in streaming sensor data, such as the abnormal data containing null values, too large values and too small values. Extracting the valuable information is essential to access the streaming data based on the characteristics of streaming data [19]. However, little attention is paid to abnormal data access. Hence, the cleaning of streaming data is rarely involved in whole data access. In general, there are two problems with existing streaming data access: (1) it is unable to handle such volumes of sensor data due to limited computing capability; (2) it is unable to achieve efficient data cleaning of streaming data with no proper data cleaning mechanism and a limited computing environment.

Apache Storm, developed by the Apache software foundation [20], is a distributed fault-tolerant and real-time streaming processing framework [21]. Storm has been effectively used for a broad variety of applications, including various data analytics, machine learning tasks, and continuous computation tasks [22]. Considering the characteristics of spatio-temporal streaming data, we proposed efficient streaming spatio-temporal data access based on Apache Storm (ESDAS) to achieve real-time streaming mass spatio-temporal vehicle data access and customized, flexible, and multi-level data cleaning. In real-time streaming mass spatio-temporal vehicle data access, the evaluation between MongoDB and ESDAS has been achieved, and the efficiency of ESDAS in speed insertion is approximately three times higher than that of MongoDB, as shown in Section 3.3. In real-time streaming mass spatio-temporal vehicle data cleaning, the filtered bus aggregation mapping with a speeding bolt, a suid bolt, a geographical position bolt, and a route bolt have been achieved in Section 3.2.

By designing an appropriate data spout/topology algorithm to access the spatio-temporal vehicle data and a comprehensive metadata model, the study achieves real time spatio-temporal data access and cleaning, achieved through experiments. In addition, the Taiyuan BD bus network is selected as the experimental network. As the consumption time of data insertion is approximately ten seconds in the current environment, which cannot meet the real-time data access demand, and valuable information is wasted, the study is meaningful and significant. The paper is organized as follows: we describe the streaming data access methodology in Section 2, where the streaming data access algorithm is also described. Experiments based on the bus data are performed, and the performance of the proposed method is evaluated in Section 3. Finally, Section 4 discusses the metrics of the proposed method and potential future directions.

## 2. Methodology

Section 2.1 describes how Apache Storm works and the architecture of ESDAS; Section 2.2 describes the table design in MongoDB for streaming raw data and filtered data for integration into Apache Storm and Sensor Observation Service (SOS); and Section 2.3 describes the interface extension of Bolt with the speeding bolt, geological bolt, suid bolt, and route bolt.

### 2.1. Apache Storm and Architecture

**Figure 1:**
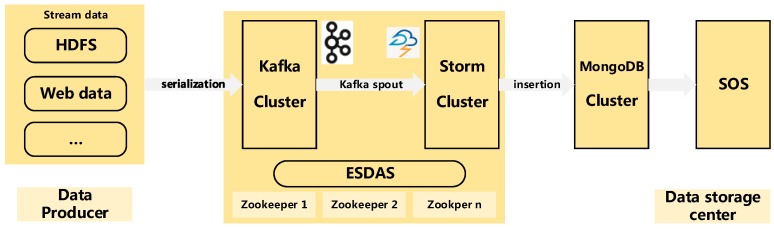
Architecture of ESDAS. Data producer refers to the Hadoop Distributed File System (HDFS), web data and other data sources; ESDAS is the data access part, containing Kafka and Storm parts, which work on Zookeeper cluster; data storage center is the MongoDB and SOS parts.

Before designing the architecture of ESDAS, there are two points to consider. One is that the architecture should consider the uninterrupted characteristic of streaming data. As the streaming data are continuous and uninterrupted, they are different from static data, so the streaming data processing tool should be applied. Another is that the architecture should be a Service Oriented Architecture (SOA) [23] so that the architecture is flexible and extendable. Figure 1 shows the architecture of ESDAS. The architecture contains three parts: a data producer, a storm layer and a data storage center. The data producer refers to the producer of streaming data. The storm layer refers to the Apache Kafka [24] and Apache Storm clusters. Storm is one of the most popular frameworks for real-time stream processing. It provides the fundamental primitives and guarantees the fault tolerance required for distributed computing in high-volume, mission-critical applications [25]. Apache Kafka is a high frequency per second distributed publish-subscribe message system, supporting Hadoop parallel data loading. In the vehicle networks, the streaming data exist in a high frequency per second manner, as the storm cluster is unstable when the streaming data exist in a high frequency per second manner. Therefore, we introduce Apache Kafka to support the streaming data serialization and data transmission in our study. The data storage center is the MongoDB cluster. MongoDB is a typical high performance NoSQL database. The SOS in ESDAS serves as a web service and receives the data insertion requests and data access requests. The Open Geospatial Consortium (OGC) defines the SOS as the one service that provides a standard application [26,27]. The programming interface manages the deployed sensors and retrieves the sensor data. So the spatio-temporal data in MongoDB can be accessed via the SOS.

A Storm cluster consists of one master node (a nimbus) and one or more worker nodes (supervisors). Spouts work as adapters that connect to a source of data, transform the data into tuples, and emit the tuples as a stream. Bolts work as the operators or functions of computation [28]. In ESDAS, there are some details of distinction. In terms of the spout/bolt design in ESDAS, the spout part and bolt part should be specified. Networks of spouts and bolts are packaged into a “topology”, which is the top-level abstraction submitted to Storm clusters for execution. A topology is a graph of stream transformations where each node is a spout or bolt. Edges in the graph indicate which bolts subscribe to which streams. When a spout or bolt emits a tuple to a stream, it sends the tuple to every bolt that subscribed to that stream. As a spout is used to emit the data, the design of ESDAS is the design of bolts. The interface of a bolt contains prepare, execute, and declareOutputFields functions. The prepare function is used to receive the data from a spout; the execute function is used to achieve a data processing procedure; and the declareOutputFields function is used to achieve the declare output of bolts. Now, the design of bolts in the topology will be described in the following sections.

**Figure 2:**
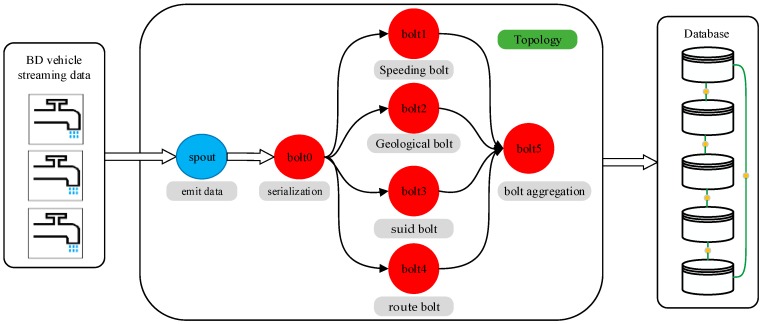
Spout/bolt workflow of topology in ESDAS.

As shown in Figure 2, the spout/bolt workflow in a topology is described as follows. Blue parts stand for the spout phase, and red parts stand for the bolt phase. The BD vehicle streaming data are the data source of the streaming data. The entrance of the topology is the spout, which is used to emit data. As the data in BD vehicle streaming data are out of order and irregular, the data are serialized in the data initialization unit. After data initialization, the data from the spout are in object sequence. In the bolt process, as shown in Figure 2, there are six bolts, from bolt0 to bolt5, including the speeding bolt, geological bolt, suid bolt, route bolt, and aggregation bolt. The speeding bolt is used to filter the speeding data in BD vehicle streaming data; the geological bolt is used to filter the BD vehicle streaming data whose geographical position is outside of Taiyuan City; the suid bolt is used to filter the BD vehicle streaming data whose bus ID is not within the reasonable ID range; and the route bolt is used to filter the BD vehicle streaming data whose bus line is outside of the common bus lines. After the processes of the speeding bolt, geological bolt, suid bolt, and route bolt, the data will aggregate in the aggregate bolt.

### 2.2. The Interface Extension of Bolt

**Figure 3:**
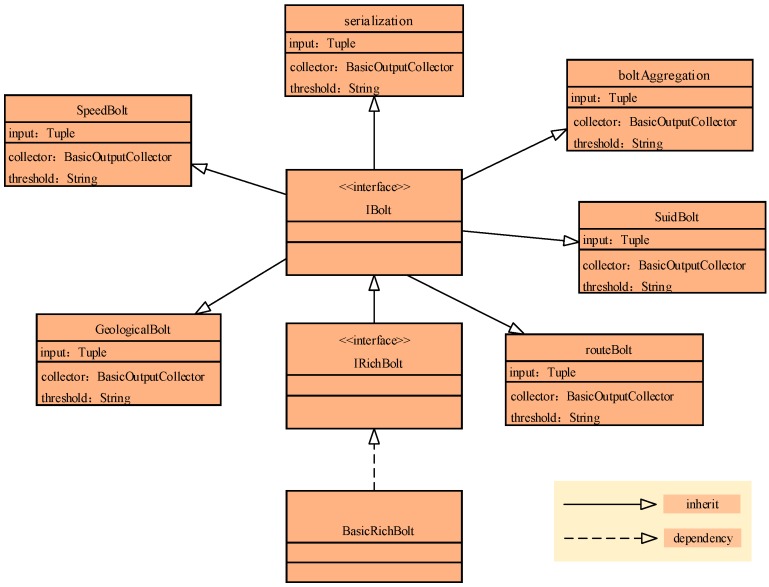
IBolt and the object extending IBolt for BD streaming vehicle data.

In speeding bolt, geological bolt, suid bolt, rout bolt, and aggregation bolt, the bolt implementation should be described. In the IBolt interface, there are three interface functions: prepare(), execute(), and cleanup(). When a worker executes the Bolt, the prepare method is first called in the context of the current execution. The execute method accepts a tuple for processing and uses the prepare method of the incoming OutputCollector ack method (expressing success) or fail (indicating failure) to feedback processing results. For the spout/bolt design in Section 2.2, the interface extension should be explained further. The prepare method remains unchanged. How to extend the execute method is essential. The filter process should be achieved in the execute method. As the filter function is the threshold comparison process, the filter function should be a judge process. For example, to filter the speeding data, the judgement “if the tuple.speed ≥30 return tuple; else {}” is made. Similar to other filter processes, these judge processes can be instantiated. As shown in Figure 3, speedBolt, GeologicalBolt, routeBolt, SuidBolt, and boltAggregation inherit the IBolt interface. The input is the Tuple element, while the parameter is the BasicOutputCollector and threshold characteristic.

### 2.3. Approach for Integrating Apache Storm with the SOS

To integrate Storm with the SOS, the data accessed from Storm should be stored in a database. In this way, the data from Storm can be accessed via SOS implementation. Therefore, the approach for integrating Storm with the SOS is the data tables designed in a database. To improve the retrieval of the BD vehicle data, we introduced the MongoDB database in our study, as shown in Figure 1. The data retrieval efficiency of MongoDB can reach several thousand times per second for the unique indexing mechanism and distributed database mechanism. In addition, MongoDB provides high efficiency of spatio-temporal data retrieval as well. In the data tables in MongoDB, there are metadata and data to store. Different from OGC implementations such as from 52° North SOS v1.0 to v4.0 [29], the data tables should consider the characteristics of streaming data. To integrate Storm with the SOS, the metadata and data should be encoded with the specified format. The SOS offers pull-based access to sensor measurements or metadata and provides standardized access to sensor observations and sensor metadata [30]. Therefore, the SOS provides the capability of accessing spatio-temporal trajectory data. The OGC provides the interface of the Observation&Measurements [31,32], which encodes the data and metadata associated with observations.

**Figure 4:**
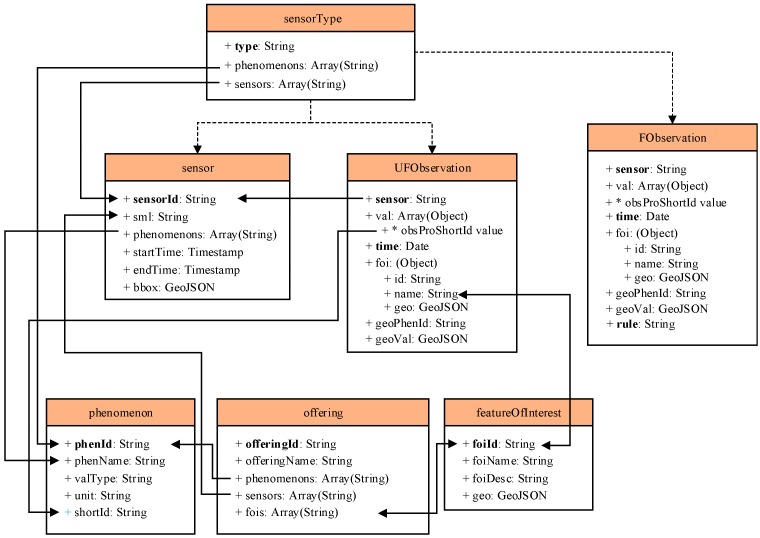
Table design in MongoDB for streaming raw data and filtered data.

As Figure 4 shows, the data tables in MongoDB contain seven sub-data tables, containing a sensorType table, a sensor table, a UFObservation table, an FObservation table, a phenomenon table, an offering table, and a featureOfInterest table. The UFObservation table is applied to store the raw data of BD streaming vehicle data, and the FObservation table is applied to store the filtered data of BD streaming vehicle data. The designs of the data tables in MongoDB for storing streaming spatio-temporal raw data, filtered data, and metadata are mainly shown as follows. The sensorType table stores the sensor type information of the sensors and phenomena; the sensor table stores the sensor information, including the sensor metadata, the time when the sensor begins observation and the time when the sensor ends observation; and the phenomenon table stores the phenomenon information, including the phenomenon name, observation value type and unit information. The offering table stores the organization information, including the organization name, phenomenon name and sensor information, and the featureOfInterest table stores the spatial arrangement of observations, including the name and coordinates encoded within the GeoJson data type. The data tables are different from others, such as the data table in The Hadoop Distributed File System [33] and are shown as follows:

(1) UFObservation table: Stores the unfiltered observation information when the sensors finish observation, including the observation time, spatial range of the observation and the observation result.
(2) FObservation table: Stores the filtered observation information when the sensors finish observation, including the observation time, spatial range of the observation and the observation result.

## 3. Experiments

Section 3.1 describes the BD buses network and the experimental environment, and Section 3.2 describes the data access result of ESDAS with different bolts such as the speeding bolt, geological bolt, suid bolt, and route bolt. Section 3.3 describes the performance evaluation of ESDAS for five thousand, ten thousand, thirty thousand, and fifty thousand records per second.

### 3.1. BD Bus Network and Experimental Environment

In our study, the BD bus network is chosen as the experimental dataset. After the U.S. global positioning system and the Russian Global Navigation Satellite System, the Chinese BD satellite navigation system is the third oldest satellite navigation system in the world. Meanwhile, the positioning accuracy of the China BD satellite navigation system is generally equal to that of GPS [34]. The TAX408BD sensor fixed on Taiyuan buses is a product module with a small volume, high sensitivity, and low power consumption that is easy to integrate. Widely used in the fields of shipping, road traffic monitoring, vehicle monitoring, vehicle navigation, handheld tracking and goods tracking, the bus sensor has features such as high precision in real time, three-dimensional positioning, three-dimensional velocity, and timing capability [34]. The inserted data sample is as follows: (lon”: “112.58497”, “lat”: “37.58712”, “suid”: “100”, “speed”: “0”, “vdesc”:“103 route number”, “plate”:” A81893”).

In our experimental environment, the distributed computing clusters have been established. In the cluster, five computers have been employed with the same configuration. The configuration of each node is an i7 4720HQ (6 M cache, eight cores, 2.60 GHz, 5 GT/s) with 8 gigabytes of Random Access Memory. They are connected to 40 GB/s InfiniBand. The Storm environment contains one nimbus node and four supervisor nodes (Worker nodes). The Nimbus is responsible for distributing code in a cluster, assigning tasks to nodes, and monitoring host failures. The Supervisor is responsible for monitoring the work of the assigned host on the work node; starting and stopping the Nimbus has been assigned to the work process. The Worker is a specific process to address Spout/Bolt logic, according to the topology parameter submitted in conf.setNumWorkers. In this way, the numbers of supervisor nodes and worker nodes are the same.

The 52° North SOS [29] is applied in the experiment. The SOS provides a data access layer through a Data Access Object paradigm. Three overwritten Data Access Object classes contain InsertObservationDAO. InsertObservationDAO can be inherited to provide the capability of accessing observations in the persistent layer. The Apache Storm 2.0 and Apache Kafka 0.8.2.0 clusters provide strong data access capabilities, and the resource consumption of the clusters is displayed in real time via the main page “http://localhost:8080”.

**Figure 5:**
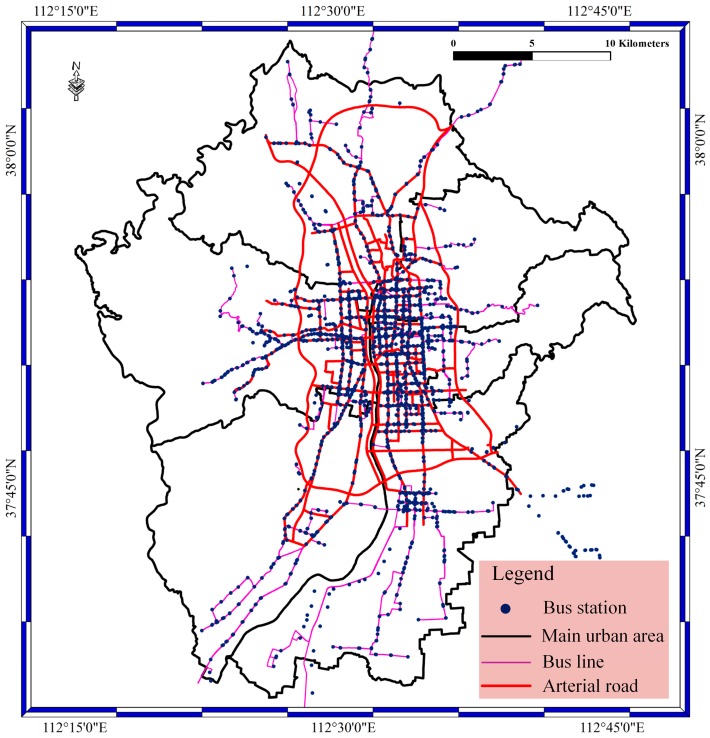
The six city areas and the spatial distributions of bus stations, bus lines and arterial roads in Taiyuan City, Shanxi Province, China.

Figure 5 shows the main city area in Taiyuan City. Red lines stand for arterial roads; the pink lines stand for bus lines; the black lines stand for the main urban areas; and the red spots stand for bus stations. The area of Taiyuan City is 1400 square kilometers, and the main city area is 400 square kilometers. The main city area of Taiyuan is surrounded by different mountains and is located on a plain of the mountains. The main city area contains six areas, the Caoping area, Xinghualing area, Wanbolin area, Yingze area, Xiaodain area, and Jingyuan area. The data scale of BD vehicle streaming data should be described in detail. The bus network in Taiyuan contains approximately 2300 buses, and the number of buses is increasing. There are approximately 800 bus stations, 400 bus lines, and 100 arterial roads in the city; each bus sends a location every 10 s; and the size of generated data is 12.67 million locations every day. The most difficult thing is to access the 2300 buses at the same time with short data latency.

### 3.2. Data Access Result with Different Bolts

**Figure 6:**
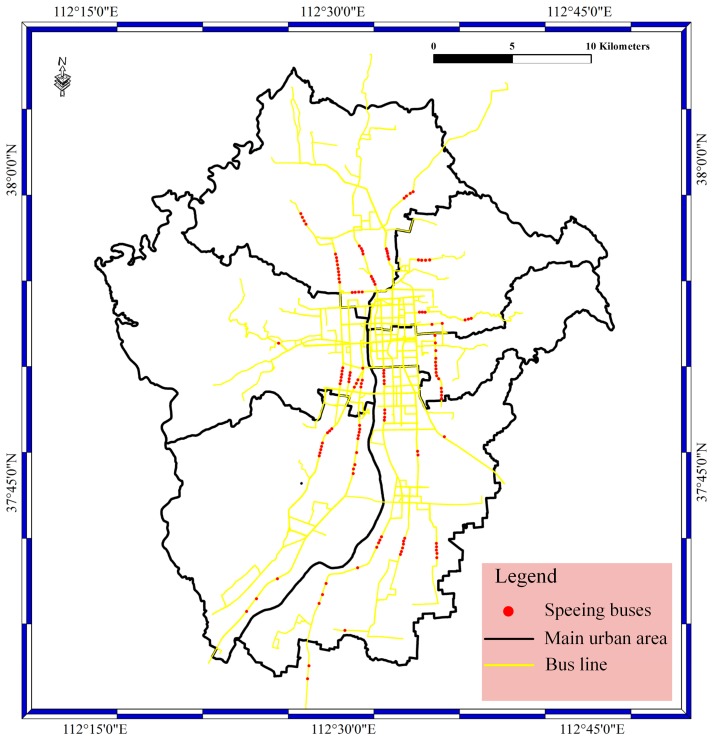
The speeding bolt result for the main urban area in Taiyuan City.

In the data access phase, BD vehicle streaming data are inserted into the Storm cluster at a high frequency and undergo data filtering. Therefore, the stored data contains the filtered data and unfiltered data. In this section, the filtered bus aggregation mapping with the speeding bolt, suid bolt, geographical position bolt, and route bolt have been achieved. In our experiments, the filtered data contain different speed data, location error data, license plate number error data, and route error data. In the speed bolt, the velocity threshold is 30 km/h, which means the bus is speeding when the velocity is greater than or equal to 30 km/h. The speeding data locations in the main urban area of Taiyuan City are shown in Figure 6. As shown in the figure, the red spots stand for the speeding buses; the yellow lines stand for bus lines; and the black lines stand for the main urban area. Most of the speeding buses are distributed in the main urban area and on the main roads in Taiyuan City.

**Figure 7:**
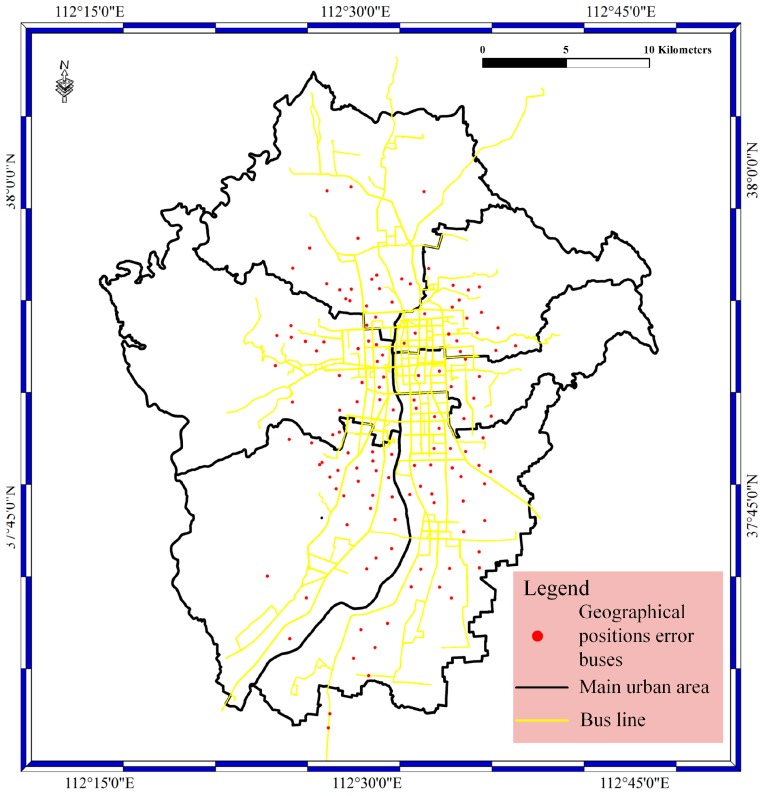
The geographical bolt result for the main urban area in Taiyuan City.

For the geological bolt, the geographical position data are filtered with the specified filter rule. Geographical position filtering is achieved by a judgment of if the geographical position is near the bus lines or near the urban area. In this bolt, the filtering occurs by judging if the distance between the geographical positions and bus lines is greater than or equal to the specified value. In our experiments, the value is set to 10 m, with consideration of system error and random error in geographical positions. In the bolt, the execute method is implemented, and the judgment phase is carried out. The prepare method is used to catch the data from the spout phase. Figure 7 shows the geographical position error results of the geological bolt phase. The red spots stand for the geographical position error of buses, as well as the geographical position error of buses located far from the main roads instead of on the main roads.

**Figure 8:**
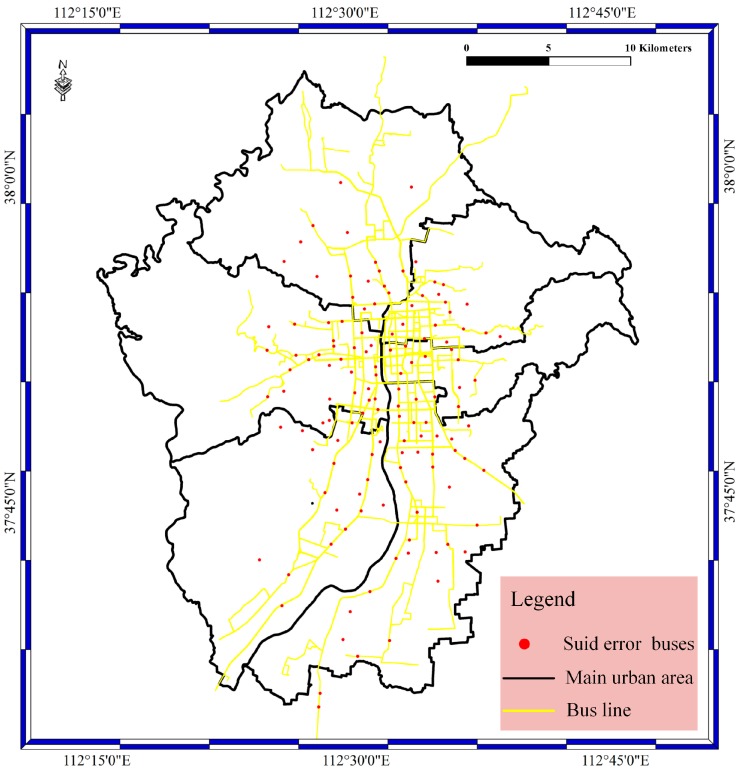
The suid bolt result for the main urban area in Taiyuan City.

For the suid bolt as shown in Figure 8, the bolt is designed to filter the BD vehicle streaming data. The suid refers to the license plate number. In the suid bolt phase, the buses without correct license plate numbers are filtered out based on whether the license plate number is in the bus license plate number database. The bus license plate number database was constructed before the experiment. In this way, the license plate number error result is determined in the suid bolt. The red spots stand for the buses with false license plate numbers. In addition, most of the buses with false license plate numbers are located on the bus lines, and a small number of spots are located in the main urban area.

### 3.3. Performance Evaluation

In the performance evaluation phase, the data access for the Taiyuan BD vehicle streaming data is evaluated. Different data sizes are evaluated in this phase, and four bolts are tested, the speeding bolt, suid bolt, geographical position bolt, and route bolt. The function of the aggregate bolt is to integrate the bolt results and insert the bolt results into the data storage center. In our experiment, the Storm and Kafka clusters are employed, and different data insertion frequencies are tested to validate the feasibility and the efficiency of the ESDAS. In this section, the performance is evaluated, and the result has been shown in Section 3.2.

**Figure 9:**
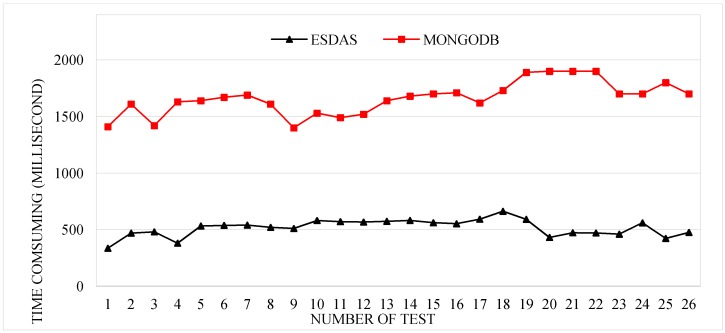
Consumption time for the speeding bolt for ESDAS and MongoDB in the ten thousand records per second case.

In this phase, we employ four kinds of Taiyuan BD bus location data with different insertion frequencies. Taken the speeding bolt test as example, we employ Taiyuan BD location buses data with five thousand, ten thousand, thirty thousand, and fifty thousand records per second. The data sample is described in Section 3.1. Data of different frequencies are inserted and filtered through ESDAS. The consumption time of the speeding bolt and MongoDB has been compared for different data volumes. Figure 9 shows the consumption times for ESDAS and MongoDB. The consumption time of ESDAS for the speeding bolt is approximately 300 milliseconds, and that for MongoDB is approximately 1300 milliseconds. The efficiency of ESDAS in the speeding bolt is approximately 3 times higher than that of MongoDB.

**Figure 10:**
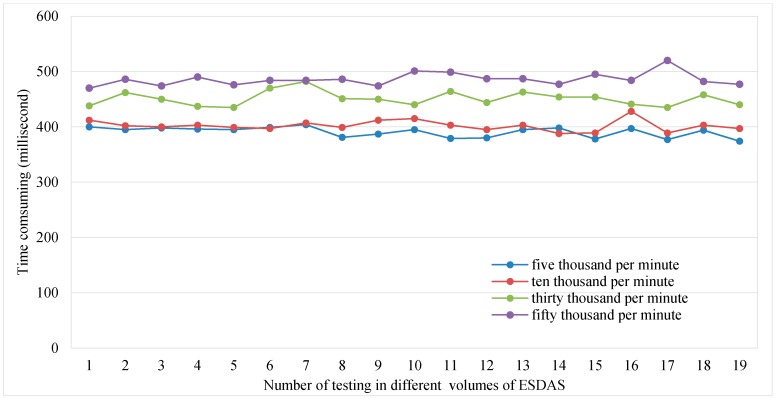
Consumption time of ESDAS for the speeding bolt in the five thousand, ten thousand, thirty thousand, and fifty thousand records per second cases.

Figure 10 shows the consumption time of ESDAS for the different data volumes of five thousand, ten thousand, thirty thousand, and fifty thousand records per second. For the different data volumes, the consumption times are different. The consumption time is evaluated approximately 20 times. The mean consumption time of ESDAS for five thousand records per second is approximately 400 ms, the consumption time for ten thousand records per second is approximately 600 ms, the consumption time for thirty thousand records per second is approximately 500 ms, and the consumption time for fifty thousand records per second is approximately 700 ms.

**Figure 11:**
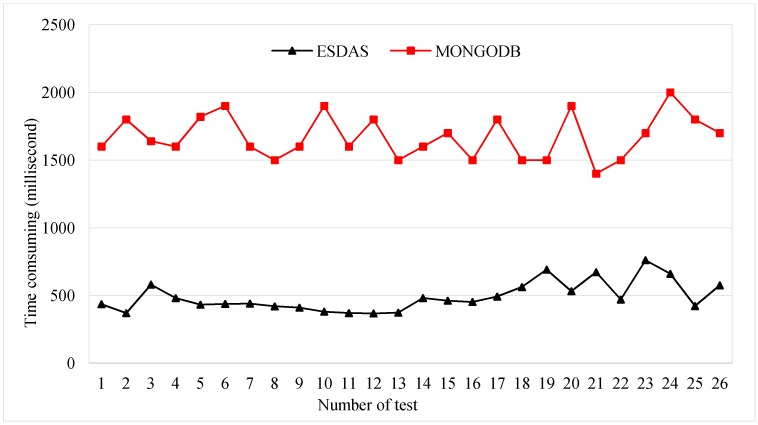
Consumption time for the suid bolt for ESDAS and MongoDB in the fifty thousand records per second case.

For the suid bolt phase, the consumption times for ESDAS and MongoDB are evaluated. The suid bolt result has been described in Section 3.2. In the five thousand, ten thousand, thirty thousand, and fifty thousand records per second cases, the consumption time is evaluated. Figure 11 shows the consumption time for the suid bolt for ESDAS and MongoDB in the ten thousand records per second case. The mean consumption time of ESDAS for the suid bolt is approximately 400 ms, and the consumption time of MongoDB is approximately 1800 ms. The efficiency of ESDAS for the suid bolt is approximately three times higher than that of MongoDB.

Similar to the speeding bolt and suid bolt, the performance evaluation of the route bolt and geological bolt can be achieved at several times. In the rest of the experiments, the consumption time for the route bolt is approximately 700 ms for fifty thousand records per second, and the consumption time for MongoDB is approximately 2400 ms. Therefore, the efficiencies for the route bolt and geological bolt are similar to those for the speeding bolt and suid bolt.

In conclusion, the data access results with different bolts have been shown in Section 3.2. The states of the filtered bus aggregations are different with different bolts. Most filtered buses for the speeding bolt are distributed on the main roads, and the most filtered buses for the geological bolt are distributed away from the roads and bus lines. For the suid bolt, most filtered buses are distributed in a uniform distribution. In terms of efficiency, the consuming time for ESDAS is much less than MongoDB in data with five thousand per second, ten thousand per second, thirty thousand per second, and fifty thousand records per second cases. Through the performance evaluation achieved in experiments, the efficiency of ESDAS in mass spatio-temporal vehicle data access is about three times higher than MongoDB.

Compared with other mass spatio-temporal data access methods as presented in Introduction Section, ESDAS has two advantages: (1) compared with direct storage methods, ESDAS can achieve real-time streaming mass spatio-temporal vehicle data access for the higher data insertion capability in five thousand per second, ten thousand per second, thirty thousand per second, and fifty thousand records per second cases as achieve in Section 3.3; (2) compared with indirect data filtering way, ESDAS can achieve customized, flexible, and multi-level data filtering as achieved in Section 3.2.

## 4. Conclusions

The streaming mass spatio-temporal data access is a hot study area in high efficient and unified access in smart city. In our study, we proposed the ESDAS method to achieve real-time streaming mass spatio-temporal vehicle data access and data cleaning as achieved in Section 3.2 and Section 3.3. In Section 3.2, the raw streaming data are made customized, flexible, and multi-level data filtering, and the filtered buses aggregation mappings with speeding bolt, suid bolt, geographical positions bolt, and route bolt have been achieved. In Section 3.3, the efficiency of ESDAS in mass spatio-temporal vehicle data access is about three times higher than MongoDB. Through integrating the SOS, the ESDAS works in web service form. The spout/bolt workflow of topology in ESDAS is illustrated in Section 2.1 and the tables design in MongoDB for streaming raw data and filtered data is described in detail. In addition, the interface extending for speeding, geological, suid, route bolts are showed in Section 2.2. The data filtering result of speeding bolt, geological bolt, and suid bolt are presented with different buses aggregation form. The efficient streaming mass data access can be applied in the traffic planning, bus feature mining, congestion prediction, and better transport management in smart city. In future work, on one hand, other bolts such as average velocity bolt, abnormal velocity bolt, maximum latitude and longitude bolt, minimum latitude and longitude and other bolts; on the other hand, other streaming data processing framework such as Apache S4 [35], Spark [36] will be applied to make the performance comparison.

## Abbreviations

The following abbreviations are used in this manuscript:

BD	BeiDou
HDFS	Hadoop Distributed File System
ESDAS	efficient streaming spatio-temporal data access based on Apache Storm
SOA	Service Oriented Architecture
OGC	Open Geospatial Consortium
SOS	Sensor Observation Service
